# Molecular Epidemiology of Carbapenem-Resistant *Acinetobacter baumannii* From Khartoum State, Sudan

**DOI:** 10.3389/fmicb.2021.628736

**Published:** 2021-02-26

**Authors:** Leena Al-Hassan, Hana Elbadawi, Einas Osman, Sara Ali, Kamal Elhag, Daire Cantillon, Julia Wille, Harald Seifert, Paul G. Higgins

**Affiliations:** ^1^Department of Global Health and Infection, Brighton and Sussex Medical School, Brighton, United Kingdom; ^2^Department of Microbiology, Soba University Hospital, University of Khartoum, Khartoum, Sudan; ^3^Faculty of Medical Laboratories, Microbiology Department, Ibn Sina University, Khartoum, Sudan; ^4^Bioscience Research Institute, Ibn Sina University, Khartoum, Sudan; ^5^College of Health Sciences, Medical Laboratory Sciences Program, Gulf Medical University, Ajman, United Arab Emirates; ^6^Institute for Medical Microbiology, Immunology and Hygiene, University of Cologne, Cologne, Germany; ^7^German Centre for Infection Research, Partner Site Bonn-Cologne, Cologne, Germany

**Keywords:** antibiotic resistance, cgMLST, OXA, NDM-1, Sudan, international clones

## Abstract

Carbapenem resistant *Acinetobacter baumannii* (CRAb) is an important global pathogen contributing to increased morbidity and mortality in hospitalized patients, due to limited alternative treatment options. Nine international clonal (IC) lineages have been identified in many countries worldwide, however, data still lacks from some parts of the world, particularly in Africa. We hereby present the molecular epidemiology of MDR *A. baumannii* from four hospitals in Khartoum, Sudan, collected from 2017 to 2018. Forty-two isolates were whole-genome sequenced, and subsequent molecular epidemiology was determined by core genome MLST (cgMLST), and their resistomes identified. All isolates had an array of diverse antibiotic resistance mechanisms conferring resistance to multiple classes of antibiotics. We found a predominance (88%) of IC2 (with the intrinsic OXA-66 and acquired OXA-23), and some with NDM-1. IC2 isolates were sub-divided into 4 STs separated by 5 to 431 allelic differences, and with evidence of seven transmission clusters. Isolates belonging to IC1, IC5, and IC9 were also identified. These data illustrate that MDR IC2 *A. baumannii* are widely distributed in Khartoum hospitals and are in possession of multiple antibiotic resistance determinants.

## Introduction

*Acinetobacter baumannii* is an important nosocomial pathogen that causes a variety of infections including but not limited to ventilator-associated pneumonia and bloodstream infection (Peleg et al., 2008). Treatment options have been compromised by the emergence of multi-drug resistant (MDR) isolates. The prevalence of MDR *A. baumannii* in hospitals has put the organism on the “ESKAPE” pathogens list: an acronym developed by the Infectious Diseases Society of America for a group of MDR nosocomial pathogens with limited remaining treatment options (Boucher et al., 2009). *A. baumannii* is also considered as priority 1 (“critical”) on the World Health Organization list of priority organisms for research and development of new antibiotics (WHO, 2017). Of particular concern is carbapenem resistance, as carbapenems are considered the drugs of last resort in treating *A. baumannii* infections, and resistance is commonly attributed to acquired carbapenemases (Poirel and Nordmann, 2006).

For the investigation of local outbreaks, pulsed field gel electrophoresis (PFGE) has been the traditional typing method, but given the global distribution of *A. baumannii*, current typing methods must be reproducible and portable, both of which PFGE lacks. Multi-locus sequence typing (MLST) is a relatively easy method to perform but it lacks the resolution for outbreak investigation (Higgins et al., 2017). The advent of relatively cheap whole-genome sequencing (WGS) technology gives the opportunity to perform high resolution typing and the ability to compare whole genomes rather than only a few loci. WGS also allows for the identification of antimicrobial resistance determinants, both intrinsic and acquired, and can aid in our understanding of the circulating resistome.

Molecular epidemiological studies have assigned nine major International Clones (IC1-9) of *A. baumannii*, the most widespread of which is IC2 commonly harboring the acquired carbapenemase OXA-23 (Higgins et al., 2010a; Zarrilli et al., 2013; Hamidian and Nigro, 2019; Müller et al., 2019; Tomaschek et al., 2019). However, there is a significant lack of epidemiological and genomic data from low- and middle-income countries (LMICs), mainly due to limited research infrastructure and resources. Given that antimicrobial resistance does not respect borders, there is therefore an urgent need to support local initiatives in LMICs to generate epidemiological and genomic data in order to assess the burden of infectious diseases, and track resistance globally (Okeke et al., 2005).

This study aimed to explore the molecular epidemiology and antimicrobial resistance mechanisms using WGS of carbapenem resistant *A. baumannii* (CRAb) isolated from patients in Khartoum State in Sudan.

## Materials and Methods

### Bacterial Strains and Antimicrobial Susceptibility

A total of 71 consecutive *A. baumannii* isolates were collected between 2017 and 2018 from hospitalized patients in four different hospitals in Khartoum, Sudan: Soba University Hospital (*n* = 37), Ibrahim Malik Hospital (*n* = 3), The Military Hospital (*n* = 16), and Al Baraha Hospital (*n* = 16). These were isolated from a variety of samples including blood, urine, sputum, wound, cerebrospinal fluid, and catheter tips (Table 1). Isolates were initially identified at the clinical microbiology laboratories of the hospitals using conventional methods. For the purpose of this study, all isolates were confirmed as *A. baumannii* by the *gyrB* multiplex PCR method and MALDI-TOF prior to whole genome sequencing (WGS) (Higgins et al., 2010b). Presence of OXA and metallo- carbapenemase-encoding genes was determined by PCR (OXA-23, -40,-51, -58, -143, -235, and NDM, IMP, VIM, GIM, KPC, and GES) (Woodford et al., 2006; Ellington et al., 2007; Higgins et al., 2013).

**TABLE 1:** Details of isolates included in the study.

						MIC mg/L									
Isolate	Genotype	Transmission cluster	Year	Specimen	Hospital	IMP	MEM	GEN	AMK	SXT	CIP	CST	ST Oxf	ST Pas	Accession No.
AC-36	IC1		2018	urine	Military	<2	≤2	≥4	≤8	≥4	≥0.5	≤2	2270	1	JABETE000000000
SUH-4	IC1		2016	blood	Soba	128	≥8	≥4	≥16	≥4	≥0.5	≤2	231	1	JABESK000000000
AC-12	IC2	TC-1	2018	sputum	Soba	64	≥8	≥4	≥16	≤2	≥0.5	≤2	195	2	JABESV000000000
AC-17	IC2	TC-1	2018	wound	Soba	64	≥8	≥4	≥16	≤2	≥0.5	≤2	195	2	JABESX000000000
AC-19	IC2	TC-1	2018	sputum	Soba	64	≥8	≥4	≥16	≤2	≥0.5	≤2	195	2	JABESY000000000
AC-2	IC2	TC-1	2018	sputum	Soba	16	≥8	≥4	≥16	≥4	≥0.5	≤2	195	2	JABESQ000000000
AC-33	IC2	TC-1	2018	sputum	Soba	64	≥8	≥4	≥16	≤2	≥0.5	≤2	195	2	JABETC000000000
AC-35	IC2	TC-1	2018	sputum	Soba	64	≥8	≥4	≥16	≤2	≥0.5	≤2	195	2	JABETD000000000
AC-37	IC2	TC-1	2018	wound	Soba	64	≥8	≥4	≥16	≤2	≥0.5	≤2	195	2	JABETF000000000
AC-9	IC2	TC-1	2018	blood	Soba	32	≥8	≥4	8	≥4	≥0.5	≤2	195	2	JABETI000000000
SUH-10	IC2	TC-1	2017	urine	Soba	256	≥8	≥4	≥16	≥4	≥0.5	≤2	208	2	JABERU000000000
SUH-12	IC2	TC-1	2017	blood	Soba	64	≥8	≥4	≥16	≤2	≥0.5	≤2	195	632	JABERX000000000
SUH-13	IC2	TC-1	2017	CSF	Soba	64	≥8	≥4	≥16	≤2	≥0.5	≤2	195	632	JABERY000000000
SUH-15	IC2	TC-1	2016	urine	Soba	128	≥8	≥4	≥16	≤2	≥0.5	≤2	208	2	JABESA000000000
SUH-20	IC2	TC-1	2017	blood	Soba	64	≥8	≥4	≥16	≤2	≥0.5	≤2	208	632	JABESC000000000
SUH-23	IC2	TC-1	2017	wound	Soba	64	≥8	≥4	≥16	≤2	≥0.5	≤2	195	632	JABESD000000000
SUH-26-1	IC2	TC-1	2017	CSF	Soba	64	≥8	≥4	≥16	≤2	≥0.5	≤2	195	632	JABESE000000000
SUH-28	IC2	TC-1	2017	wound	Soba	64	≥8	≥4	≥16	≥4	≥0.5	≤2	208	2	JABESH000000000
SUH-3	IC2	TC-1	2016	blood	Ibrahim Malik	64	≥8	≥4	≥16	≤2	≥0.5	≤2	208	2	JABESJ000000000
SUH-5	IC2	TC-1	2016	catheter	Soba	256	≥8	≥4	≥16	≤2	≥0.5	≤2	208	2	JABESL000000000
SUH-8	IC2	TC-1	2016	urine	Soba	256	≥8	≥4	≥16	≥4	≥0.5	≤2	195	2	JABESO000000000
SUH-9	IC2	TC-1	2017	blood	Soba	64	≥8	≥4	≥16	≤2	≥0.5	≤2	195	632	JABESP000000000
SUH-29	IC2		2019	blood	Soba	64	≥8	≥4	≥16	≤2	≥0.5	≤2	1632	600	JABESI000000000
AC-5	IC2	TC-2	2018	blood	Military	16	≥8	≥4	≥16	≤2	≥0.5	≤2	208	2	JABESS000000000
AC-7	IC2	TC-2	2018	sputum	Military	32	≥8	≥4	≥16	≥4	≥0.5	≤2	208	2	JABESU000000000
AC-3	IC2	TC-3	2018	sputum	Military	16	≥8	≥4	≥16	≤2	≥0.5	≤2	208	2	JABESR000000000
AC-6	IC2	TC-3	2018	sputum	Military	32	≥8	≥4	≥16	≤2	≥0.5	≤2	208	2	JABEST000000000
SUH-11-1	IC2	TC-4	2017	blood	Ibrahim Malik	64	≥8	≥4	≥16	≥4	≥0.5	≤2	208	2	JABERV000000000
SUH-11-2	IC2	TC-4	2017	blood	Ibrahim Malik	64	≥8	≥4	≥16	≥4	≥0.5	≤2	208	2	JABERW000000000
SUH-26-2	IC2	TC-4	2017	CSF	Soba	64	≥8	≥4	≥16	4	≥0.5	≤2	195	2	JABESF000000000
AC-26	IC2	TC-5	2018	sputum	Soba	64	≥8	≥4	≥16	≤2	≥0.5	≤2	1632	600	JABETB000000000
AC-27	IC2	TC-5	2018	sputum	Soba	128	≥8	≥4	≥16	≤2	≥0.5	≤2	1632	600	JABETJ000000000
SUH-6	IC2	TC-5	2016	wound	Soba	256	≥8	≥4	≥16	≤2	≥0.5	≤2	1632	600	JABESM000000000
SUH-27	IC2	TC-6	2017	wound	Soba	64	≥8	≥4	≥16	≥4	≥0.5	≤2	208	2	JABESG000000000
SUH-7	IC2	TC-6	2016	wound	Soba	64	≥8	≥4	≥16	≥4	≥0.5	≤2	195	2	JABESN000000000
AC-14	IC2	TC-7	2018	sputum	Al-Braha	128	≥8	≥4	≥16	≤2	≥0.5	≤2	1701	570	JABESW000000000
AC-23	IC2	TC-7	2018	catheter	Al-Braha	128	≥8	≥4	≥16	≤2	≥0.5	≤2	1701	570	JABETA000000000
AC-40	IC2	TC-7	2018	sputum	Al-Braha	64	≥8	≤2	≥16	≤2	≥0.5	≤2	1701	570	JABETG000000000
AC-45	IC2	TC-7	2018	sputum	Al-Braha	128	≥8	≥4	≥16	≤2	≥0.5	≤2	1701	570	JABETH000000000
SUH-14	IC5		2016	urine	Soba	128	≥8	≥4	≥16	≥4	≥0.5	≤2	732	602	JABERZ000000000
AC-20	IC9		2018	tissue	Military	128	≥8	≥4	≥16	≤2	≥0.5	≤2	1089	85	JABESZ000000000
SUH-2	S		2016	wound	Soba	4	≤2	≥4	≤8	≤2	≥0.5	≤2	1208	584	JABESB000000000

*Isolate number, genotype, transmission clusters, year of collection, type of specimen, hospital from which it was collected, MIC, ST (Oxford and Pasteur schemes), and GenBank accession numbers. IC, international clone; S, singleton, TC, transmission cluster; CSF, cerebrospinal fluid; IMP, imipenem; MEM, meropenem; GEN, gentamicin; AMK, amikacin; SXT, trimethoprim/sulfamethoxazole; CIP, ciprofloxacin; CST, colistin. Some isolates have two different STs in the Oxford scheme due to two *gdhB* alleles detected, we give only the first one. SUH-29 was not part of a transmission cluster. AC-36 contained a novel *gpi* allele (assigned number 388), and subsequently a novel ST was assigned: ST-2270 in the Oxford MLST scheme.*

Antimicrobial susceptibility (AST) was initially performed by disk-diffusion at the clinical microbiology laboratories and interpreted according to CLSI guidelines. Upon species confirmation, the minimum inhibitory concentration (MIC) was determined by MICRONAUT-GN, an automated microtiter broth dilution susceptibility testing system (Merlin Diagnostika, Germany). Colonies from a pure overnight subculture were used to prepare a 0.5-McFarland-standard suspension in 0.9% saline as recommended by the manufacturer (Merlin Diagnostika, Germany). This system allows the determination of the MIC for a panel of antibiotics with two concentrations (mg/L) based on the EUCAST MIC breakpoints for sensitivity (S) or resistance (R) (EUCAST, 2020b): meropenem (MER), gentamicin (GEN), amikacin (AMK), trimethoprim-sulfamethoxazole (SXT), ciprofloxacin (CIP), and colistin (COL) in a single microtitre plate. After the addition of the bacterial suspension and rehydration of the antibacterial agents, the plates were incubated at 37°C for 18–24 h. Bacterial growth in the wells was monitored photometrically at 620 nm, and a density of >50% above the cutoff value for the growth control. The results were also observed visually for turbidity to confirm the results. Additionally, MIC for imipenem (IMP) was performed by broth microdilution (BMD) method according to the EUCAST guidelines V2, 2020 (EUCAST, 2020a). Quality control strains *Escherichia coli* ATCC 25922 and *Pseudomonas aeruginosa* ATCC 27853 were used in all AST experiments.

### Whole Genome Sequencing and Molecular Typing

Total DNA was extracted from the bacterial isolates using the MagAttract HMW DNA Kit (Qiagen, Hilden, Germany) according to the manufacturer’s instruction. Sequencing libraries were prepared using the Nextera XT library prep kit (Illumina GmbH, Munich, Germany) for a 250-bp paired-end sequencing run on an Illumina MiSeq platform. Genomes were assembled *de novo* using Velvet v1.1.04. Molecular epidemiology of the isolates was investigated by core-genome MLST (cgMLST) based on a core genome of 2,390 alleles using Ridom < *cps*:*sup* > ® < /*cps*:*sup* > SeqSphere+ v. 7.2.3 (Higgins et al., 2017).

Multi-locus sequence typing was performed on the pubMLST database https://pubmlst.org/abaumannii/ to identify sequence types (STs) for both the Pasteur and Oxford schemes. Antimicrobial resistance determinants were identified using the Resfinder software v3.2 https://cge.cbs.dtu.dk/services/ResFinder/. Beta-lactamases were identified using the online beta-lactamase database^1^ (Naas et al., 2017).

All assembled genomes were submitted to GenBank under BioProject number PRJNA628907. All accession numbers are listed in Table 1.

## Results

Of the 71 isolates initially collected, a total of 42 isolates were confirmed as *A. baumannii* by *gyrB*, presence of *bla*_OXA–51–like_, and MALDI-TOF, and subsequently included in this study. The remaining isolates were other Gram negative organisms misidentified as *A. baumannii* and excluded from the study.

Antimicrobial susceptibility results showed that all isolated were MDR (Table 1) and that 95% of the isolates (*n* = 40) were CRAb, with an MIC for imipenem ranging from 16 to 256 mg/L (Table 2). Resistome analysis revealed that *bla*_OXA–23_ was the most prevalent carbapenemase, present in 39 isolates (92%), with three isolates co-harboring *bla*_OXA–58_ (Table 2). Other β-lactamases detected included NDM-1 (*n* = 10), GES-11 (*n* = 2), CTX-M-15 (*n* = 1), OXA-1 (*n* = 1), TEM-1D (*n* = 30) and TEM-199 (*n* = 1). Multiple aminoglycoside modifying enzymes were identified: *armA*, *aadA1*, *aad-B-like*, *aac(3)-Ia-like*, *aph(3′)-VIa-like*, *aph(3′)-Ia*, *aph(6)-Id*, *aph(3″)-Ib, aph(3′)-Ic, strA*, *strB*, and *aac(6′)-Ib-like*. Genes contributing to macrolide resistance *mphE* and *msrE* were present in 33 isolates, some of which co-localized in a gene cassette with aminoglycoside resistance genes. Twenty-five isolates possessed *tet(A)*, *tet(B)* or *tet(39)* contributing to tetracycline and erythromycin resistance, found on the same contigs as aminoglycoside modifying enzymes. All isolates had the chromosomally encoded ADC cephalosporinase.

**TABLE 2:**
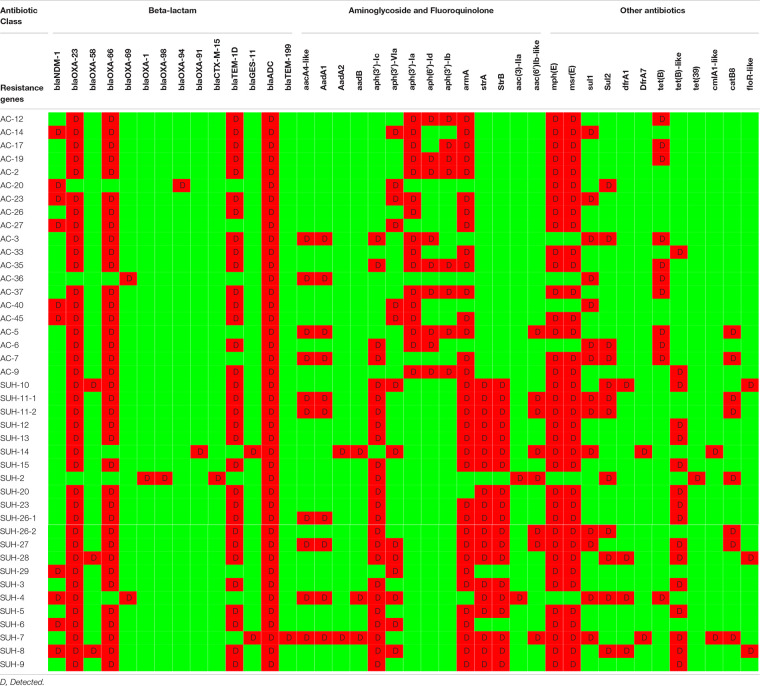
Resistome data for all isolates.

The isolates belonged to seven distinct STs according to the Pasteur scheme: ST1, ST2, ST85, ST570, ST584, ST600, ST602, and ST632, which belonged to IC1, IC2, IC5, IC9, and a singleton. Table 1 lists the STs identified in both the Pasteur and Oxford schemes. OXA-66, OXA-69, OXA-91, OXA-94, and OXA-51 were the intrinsic OXA-51-like enzymes identified in the study (Table 2).

**FIGURE 1:**
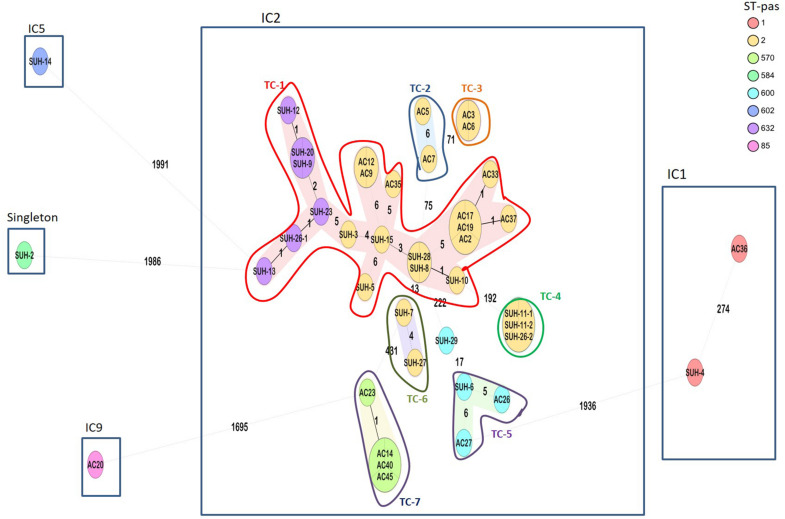
Ridom SeqSphere+ minimum spanning tree (MST) for 42 samples based on 2,390 alleles. Isolates grouped by color indicating the different STs (Pasteur scheme). Eight different STs were identified, belonging to IC1, IC2, IC5, IC9, and a singleton. Numbers between the nodes indicate the number of allelic differences. Shaded nodes represent transmission clusters (TC), and are numbered TC1-7.

Molecular epidemiology by cgMLST confirmed that IC2 with the intrinsic OXA-66 and acquired OXA-23 was the major clonal lineage found across the four hospitals in Khartoum (*n* = 37, 88%). As seen in Figure 1, IC2 is sub-divided into 4 STs (ST-2, −570, −600, and −632) separated by 5–431 allelic differences. Within IC2, we found evidence of seven transmission clusters (TC) (defined at ≤10 allelic differences between isolates) one of which included two Pasteur STs (ST-632 and ST-2), including two inter-hospital transmissions (Figure 1). The largest transmission cluster (TC-1, *n* = 20) comprises two Pasteur STs: ST-2 and ST-632 (single-locus variants at the *rpoB allele*), and two Oxford STs: ST-195 and ST-208 (single locus variants at the *gpi* allele).

Two isolates belonging to IC1 (ST-1) and harboring OXA-69 were from two different hospitals (Soba and the Military Hospital), separated by >200 allelic differences, and with distinct resistomes. The two isolates shared the presence of aminoglycoside modifying enzymes *aadA1* and *aac(3)-Ia*, and *sul1*. Isolate SUH-4 was carbapenem resistant, and harbored OXA-23, NDM-1, and additional aminoglycoside modifying enzymes *aadB-like, aph(3′)VIa, aph(3′)Ic, strA, strB*, and *dfrA1* contributing to trimethoprim resistance. Isolate AC-36, on the other hand, was carbapenem susceptible.

The remaining isolates SUH-14 (OXA-91, ST-602) and AC-20 (OXA-94, ST-85) belonged to IC5 and IC-9, respectively, while SUH-2 (OXA-98, ST-584) was a singleton not belonging to an IC.

These data also gave an indication of the local inter-hospital transmissions. Soba University Hospital had a large diversity of isolates belonging to IC1, IC2, IC5 and the singleton ST-584. As seen in Figure 1, the majority of these isolates were part of a large transmission cluster (TC-1) that included an isolate from the Ibrahim Malik hospital, as well as having three other smaller transmission clusters (TC-4, TC-5, and TC-6), of which TC-4 comprised three identical isolates, two of which were from the Ibrahim Malik hospital. Isolates from the other two hospitals, Al-Baraha and the Military Hospital, in this study were part of transmission clusters TC-2, TC-3, and TC-7, but with no inter-hospital spread.

With regards to carbapenem-resistance, all but three isolates possessed *bla*_OXA–23_ which was the sole carbapenemase in 26 isolates. In addition to *bla*_OXA–23,_ two isolates had *bla*_GES–11_, eight *bla*_NDM–1_, two *bla*_OXA–58_, and one had both *bla*_NDM–1_ and *bla*_OXA–58_ (Table 2 and Supplementary Figure 2). One isolate had only NDM-1. There were two carbapenem susceptible isolates, one of which was positive for the ESBLs CTX-M-15 and *bla*_OXA–1_. The plasticity of the isolates is demonstrated by them being identical by cgMLST, but differing in their resistomes as for of isolate SUH-8 differing from SUH-28 by having an additional *bla*_NDM–1_.

Three isolates from Ibrahim Malik Hospital all fall within IC2 (ST-2) but one of which was separated from the other two by >192 allelic differences. The Military Hospital, on the other hand, had isolates belonging to IC1, IC2, and IC9. A single cluster represented isolates from Al-Baraha Hospital (ST-570) belonging to IC2 and all co-harbored OXA-23 and NDM-1.

## Discussion

We hereby present the molecular epidemiology of carbapenem-resistant *A. baumannii* collected from patients from Khartoum, Sudan. A total of 71 isolates from four different hospitals were collected in 2017–2018, out of which 42 were confirmed as *A. baumannii*. Routine identification in clinical microbiology laboratories in Sudan relies primarily on phenotypic methods in combination with simple biochemical methods, which are not always reliable for accurate bacterial species identification, and 29 isolates (40%) were excluded in the study due to misidentification. Similar findings were reported in a study in Sudan on *Klebsiella pneumoniae* where 40% of isolates were misidentified (Osman et al., 2020). These data highlight an urgent need to improve diagnostic facilities across LMICs such as Sudan, in order to obtain accurate information of the local burden of infections and resistance, and to select optimal treatment options. However, restrictions in funding and capacity pose great limitations in implementing molecular diagnosis in clinical microbiology laboratories. Genomic data from Sudan is scarce, despite the indicative high burden of MDR in hospitals (Hamdan et al., 2015). One study that has performed WGS on an *A. baumannii* isolate from Khartoum, identified it as ST^OXF^164, with OXA-91 as the intrinsic OXA-51-like enzyme (Mohamed et al., 2019), however, the genome of this isolate had over 2,000 allelic differences when compared to our isolates (data not shown).

Core-genome MLST analysis revealed seven transmission clusters, with a predominance of IC2-OXA-23 accounting for 88% of the isolates across Khartoum Hospitals. This is in accordance with previously published data showing that IC2-OXA-23 is the most successfully disseminating clone globally (Hamidian and Nigro, 2019), however, there are regional variation such as IC5 and IC7 being more predominant in South and Central America (Higgins et al., 2010a). IC2 in Sudan is composed of four sequence types (ST^PAS^2, ST^PAS^570, ST^PAS^600, and ST^PAS^632). The IC2 isolates were further delineated by cgMLST showing that the ST2 isolates were in some cases more distant than to other STs. For example >190 allelic differences are found between isolates within ST2 (SUH-10 and SUH-11), whereas SUH-3 and SUH-23 which belong to ST2 and ST632, respectively, only have 5 allelic differences. A recent comparison of the two MLST schemes suggested that the Pasteur scheme was more stable than the Oxford scheme (Gaiarsa et al., 2019); while this might be true in most cases, these data from Sudan highlight that a reliance on only 7-loci can lead to a false sense of strain divergence, when in fact, isolates differ in five alleles out of a total 2,390, one of which is the *rpoB* MLST allele. Furthermore, the seven transmission clusters, included inter-hospital spread of two clones (TC-1 and TC-4), which was not apparent from their STs. Similarly, isolates belonging to ST1 have >200 allelic differences between them. These findings illustrate the low resolution of traditional 7-loci typing, particularly for local outbreak and transmission investigations.

Although transmission clusters are clearly evident (Figure 1), and despite the relatively conserved dissemination of carbapenemases in the current study, there are some differences, particularly in acquired genes such as NDM-1. For example, SUH-28 and SUH-8 were identical by cgMLST and both had OXA-23, but SUH-28 harbored additional NDM-1 and OXA-58. Furthermore, NDM-1 was present in only three of the four ST-600 isolates. Relying on characterisation of the resistome for determining clonality can be misleading and illustrate the genome plasticity of MDR *A. baumannii*. It is therefore important that discriminatory genomic typing methods are applied in combination with the resistome, clinical and epidemiological data to obtain an accurate picture of outbreaks and transmission links. As *A. baumannii* is an emerging nosocomial pathogen with extended antibiotic resistance, it’s important to note the availability of online resources and databases offering rapid typing and phylogenetic relatedness to use when investigating local and global outbreaks, which is increasingly important in a globalized community.

Three isolates (SUH-2, AC20 and AC36) did not harbor OXA-23, but on the other hand harbored other β-lactamases: CTX-M-15, OXA-1, and/or NDM-1. To our knowledge, this is the first report of OXA-1 in *A. baumannii*. OXA-1 is commonly found in *Enterobacteriaceae*, and frequently co-carried with CTX-M-15, as also present in our data (Sugumar et al., 2014).

Our study complements previous studies on the successful dissemination and possible endemicity of specific STs and resistance determinants across North Africa and the Middle East. ST-85 (IC9) is a common clone in Africa and the Middle East as it has been reported as a major clone spreading the NDM-1 gene in Tunisia, among Syrian refugees in Lebanon and Turkey, in France from patients with a history of hospitalization in Algeria, Tunisia and Egypt (Bonnin et al., 2013; Jaidane et al., 2018; Salloum et al., 2018), and recently Spain in a strain harboring NDM-6 (Xanthopoulou et al., 2020). ST6, a single locus variant of ST85, has also been identified among Lebanese patients (Rafei et al., 2014). A single isolate from the Military Hospital was identified as ST-85 harboring OXA-94 and NDM-1 similar to the isolates from Tunisia and Lebanon. With Oxford MLST scheme, this isolate (AC20) was assigned to ST-1089^OXF^, which has also been reported in Egypt (Al-Hassan et al., 2019; Al-Hassan and Al-Madboly, 2020). Two isolates, SUH-14 and SUH-7, assigned to ST602 (IC5) and ST-2 (IC2), respectively, were positive for GES-11 ESBL. GES-11 has been identified in a Belgian outbreak and was found associated with travel to Egypt, Turkey, and Gaza, thereby indicating a possible geographic dissemination (Moubareck et al., 2009; Bogaerts et al., 2010). Travel and medical tourism are among the major contributors to the acceleration of spread of resistance globally (Ostholm-Balkhed et al., 2013; Tatem, 2014).

## Conclusion

Our study illustrates that CRAb IC2 *A. baumannii* are widely distributed in Khartoum hospitals and are in possession of multiple antibiotic resistance determinants. We are also able to confirm the spread of specific clones, such as IC9 (ST-85) globally, and across the Middle East and North Africa region specifically. Despite advances in genomic technology, and the relative cheap price of conducting WGS on bacterial genomes, it remains a challenge to implement routine molecular typing methods in Sudan, particularly due to the need of advanced technology and expertise. It is, however, essential to support local efforts to obtain epidemiological data on the burden of resistance in major hospital-acquired infections such as those caused by *A. baumannii*. For local outbreak investigations, molecular epidemiology must be combined with patient clinical and demographic data in order to track transmission and resistance.

## Data Availability Statement

The data presented in the study are deposited in GenBank, under BioProject PRJNA628907. All accession numbers are listed in Table 1.

## Author Contributions

LA-H, HE, EO, DC, and PH contributed to the design of the experiments. HE, EO, SA, and KE were responsible for the collection of isolates from the hospitals. LA-H, HE, EO, DC, and JW performed the experiments. LA-H, HE, EO, JW, HS, and PH analyzed and interpreted the data. LA-H, HE, and PH wrote the manuscript. All authors contributed to the article and approved the submitted version.

## Conflict of Interest

The authors declare that the research was conducted in the absence of any commercial or financial relationships that could be construed as a potential conflict of interest.

## Footnotes

1 http://www.bldb.eu/

## Supplementary Material

Supplementary Figure 1 Ridom SeqSphere+ minimum spanning tree (MST) for 42 samples based on 2,390 alleles, Numbers between the nodes indicate the number of allelic differences. Isolates grouped based on the hospital they were collected from.

Supplementary Figure 2 Ridom SeqSphere+ minimum spanning tree (MST) for 42 samples based on 2,390 alleles. Color of nodes indicate the different β-lactamases they harbor. Numbers between the nodes indicate the number of allelic differences. OXA-23 is the main carbapenemase. NDM-1 is present in 10 isolates representing IC1, IC2, and IC9. Several isolates harbor multiple β-lactamases.
