# Supplementary figures and images for: Molecular Epidemiology of Carbapenem-Resistant Acinetobacter baumannii From Khartoum State, Sudan
Source: Front Microbiol. 2021 Feb 26;12:628736. doi: 10.3389/fmicb.2021.628736 (PMC7952628; doi:10.3389/fmicb.2021.628736)

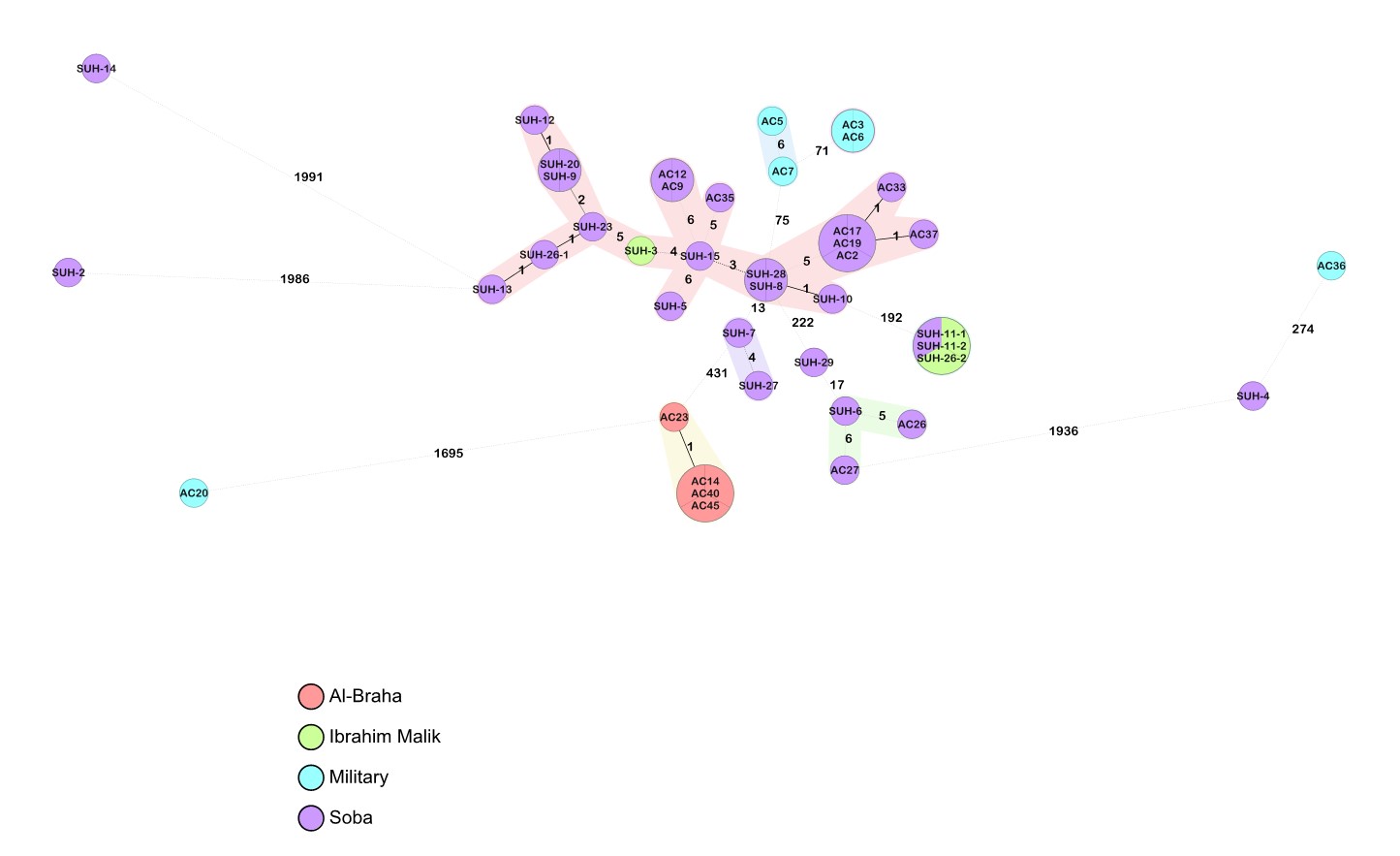

Supplement: Supplementary Figure 1 — Ridom SeqSphere+ minimum spanning tree (MST) for 42 samples based on 2,390 alleles, Numbers between the nodes indicate the number of allelic differences. Isolates grouped based on the hospital they were collected from. [file Image_1.JPEG]

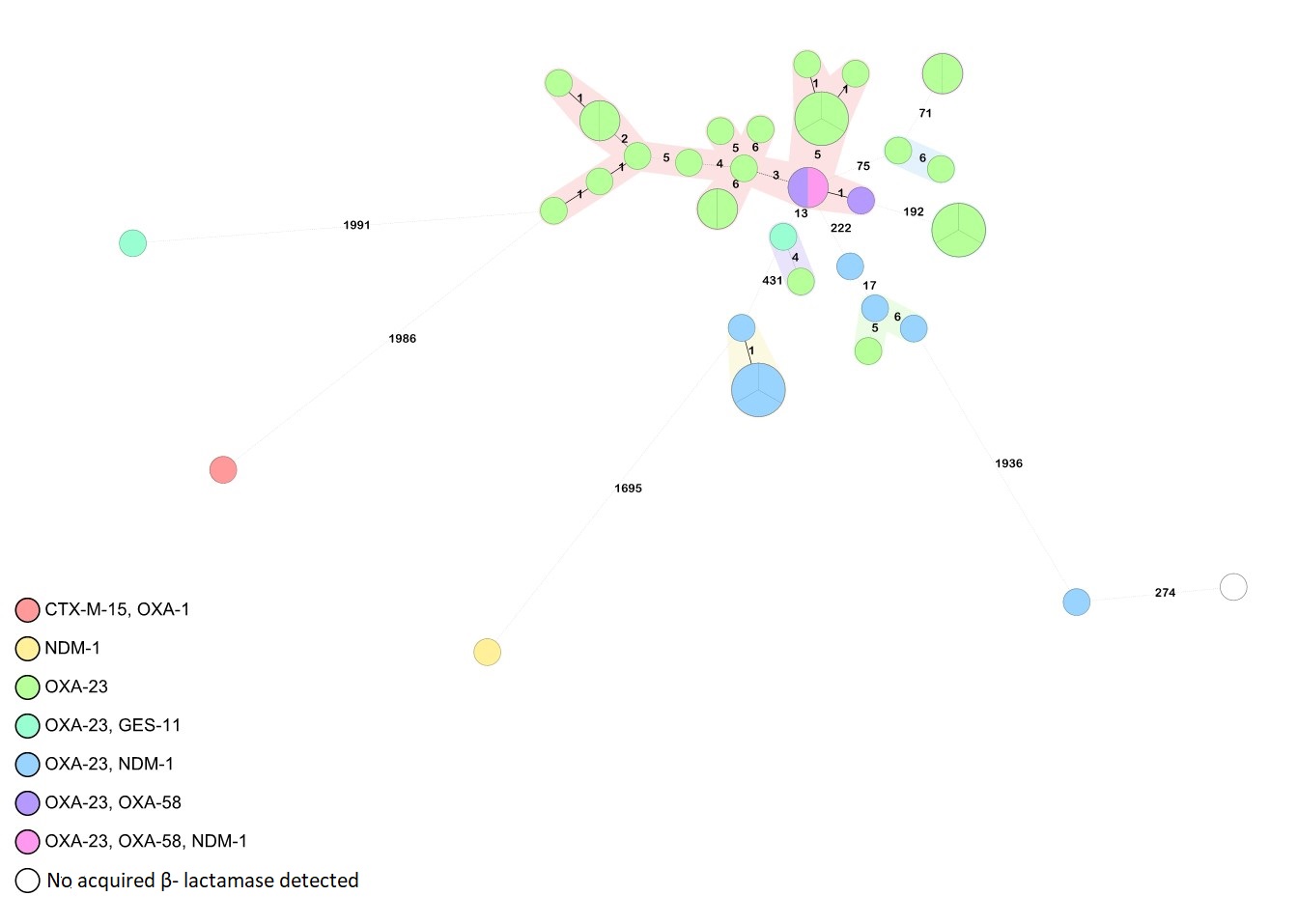

Supplement: Supplementary Figure 2 — Ridom SeqSphere+ minimum spanning tree (MST) for 42 samples based on 2,390 alleles. Color of nodes indicate the different β-lactamases they harbor. Numbers between the nodes indicate the number of allelic differences. OXA-23 is the main carbapenemase. NDM-1 is present in 10 isolates representing IC1, IC2, and IC9. Several isolates harbor multiple β-lactamases. [file Image_2.JPEG]
